# Emerging Opportunity and Destiny of *mcr-1*- and *tet*(X4)-Coharboring Plasmids in Escherichia coli

**DOI:** 10.1128/Spectrum.01520-21

**Published:** 2021-12-08

**Authors:** Xiaoyu Lu, Xia Xiao, Yuan Liu, Ruichao Li, Zhiqiang Wang

**Affiliations:** a Jiangsu CoInnovation Center for Prevention and Control of Important Animal Infectious Diseases and Zoonoses, College of Veterinary Medicine, Yangzhou University, Yangzhou, Jiangsu Province, People’s Republic of China; b Institute of Comparative Medicine, Yangzhou University, Yangzhou, Jiangsu Province, People’s Republic of China; Forschungszentrum Jülich GmbH

**Keywords:** *Escherichia coli*, *tet*(X4), *mcr-1*, fusion plasmids, plasmid evolution, fitness costs

## Abstract

The emergence of the plasmid-mediated colistin resistance gene *mcr-1* and the plasmid-mediated tigecycline resistance gene *tet*(X4) represents a significant threat to public health. Although *mcr-1* and *tet*(X4) have been reported to coexist in the same isolate, there are no reports on the emergence of plasmids coharboring *mcr-1* and *tet*(X4). In this study, we aimed to investigate the opportunities for the emergence of *mcr-1-* and *tet*(X4)-coharboring plasmids and their destiny in Escherichia coli. Two plasmids carrying both *mcr-1* and *tet*(X4) were constructed through conjugation assays and confirmed by S1 nuclease pulsed-field gel electrophoresis (S1-PFGE) and Nanopore long-read sequencing. Seven evolved plasmids carrying *mcr-1* and *tet*(X4) from one of the two plasmids were acquired after continuous evolutionary processes. The fitness effects of *mcr-1-* and *tet*(X4)-coharboring plasmids were studied by stability experiments, competition experiments, and growth curve measurements. A plasmid carrying *mcr-1* and *tet*(X4) and conferring no fitness cost to its host strain E. coli C600 emerged after evolution during serial passages of bacteria. We proved that it can be anticipated that *mcr-1* and *tet*(X4) could appear in a single plasmid, and the possibility of occurrence in field strains should be monitored constantly. The originally formed cointegrate plasmids coharboring *mcr-1* and *tet*(X4) could evolve into a plasmid with lower fitness costs. This will undoubtedly accelerate the transmission of *mcr-1* and *tet*(X4) globally. The findings highlighted the great possibility of novel hybrid plasmids positive for *mcr-1* and *tet*(X4), and the risk is worthy of increasing attention and public concern globally.

**IMPORTANCE** Tigecycline and colistin are used as last-resort therapies to treat infections caused by multidrug-resistant (MDR) Gram-negative bacteria. However, the emergence of the plasmid-mediated tigecycline resistance gene *tet*(X4) and the plasmid-mediated colistin resistance gene *mcr-1* represents a significant threat to human health. A plasmid coharboring *mcr-1* and *tet*(X4) has not emerged so far, but the potential risk should not be ignored. Plasmids coharboring such vital resistance genes will greatly accelerate the progression of pan-drug resistance among pathogens globally. Therefore, evaluation of the emerging opportunity for the *mcr-1-* and *tet*(X4)-coharboring plasmids and their destiny in E. coli is of great significance. We provide important insight into the contributions of *intI1*, IS*26*, a truncated IS*CR2* (ΔIS*CR2*), and IS*4321*R during the generation of cointegrate plasmids carrying *mcr-1* and *tet*(X4) and highlight the importance of antimicrobials in the evolution and diversity of *mcr-1-* and *tet*(X4)-coharboring plasmids. We show that monitoring of the occurrence of *mcr-1*-carrying MDR plasmids and *tet*(X4)-bearing MDR plasmids in the same strain should be strengthened to avoid the formation of *mcr-1-* and *tet*(X4)-coharboring plasmids.

## INTRODUCTION

Infections caused by antimicrobial-resistant pathogens kill hundreds of thousands of people every year worldwide. Colistin and tigecycline are used as last-resort therapies to treat infections caused by multidrug resistant (MDR) Gram-negative bacteria, especially carbapenem-resistant *Enterobacteriaceae* (CRE) (1–3). However, plasmid-mediated colistin resistance genes (*mcr-1* to *mcr-10*) have been reported all over the world since the first report of *mcr-1* in 2016 (4, 5). *mcr-1* is still the most widespread *mcr* gene and has been identified from various sources. The IncHI2 plasmid is one of the most prevalent *mcr-1*-bearing plasmid types, and IncHI2 plasmids are usually MDR plasmids carrying various resistance genes and insertion sequences (ISs) (6, 7), especially IS*26*, which is related to cointegration of plasmids (8, 9). Importantly, the IncHI2 plasmids were also important vectors for *bla*_NDM-5_, *bla*_CTX-M-65_, and *bla*_OXA-1_, which were great threats to public health (10, 11). Plasmid-mediated tigecycline resistance genes *tet*(X3) and *tet*(X4) were discovered in China in 2019 (12, 13). Since then, diverse *tet*(X) genes, *tet*(X5) to *tet*(X14), have been described (14). *tet*(X4) was the most widespread *tet*(X) variant. Most *tet*(X4)-positive plasmids are MDR plasmids with various replicon types (e.g., IncFII, IncX1, IncFIB, IncFIA, InHI1, and IncA/C2) carrying various ISs, especially IS*26* and IS*CR2* (15). IS*CR2* was adjacent to *tet*(X4) in most plasmids and played a role in facilitating the transmission of *tet*(X4) (12, 13). We reported previously that IS*CR2* played an important role in facilitating the generation of *mcr-1*-bearing, large, fused, MDR plasmids (16).

The emergence of *mcr-1-* and *tet*(X4)-cocarrying strains was worrisome, as the bacteria could be resistant to two last-resort antimicrobials, tigecycline and colistin. An increasing number of articles in the literature are reporting on the coexistence of *mcr-1* and *tet*(X4) in one isolate but on two different plasmids (17–19). Xu et al. reported the cocarriage of *tet*(X6) and *mcr-1* by a single plasmid in Escherichia coli (20). Sun et al. reported the coexistence of *tmexCD1-toprJ1* with *mcr-8* in one plasmid in Klebsiella pneumonia (21). Fortunately, a plasmid coharboring *mcr-1* and *tet*(X4) has not emerged so far, since once such a plasmid appears, it will greatly promote the explosion of global pan-drug-resistant bacteria. Because of the close relationship between ISs and MDR cointegrate plasmids, we anticipated that under the mediation of ISs, *mcr-1* and *tet*(X4) may appear in the same fusion/cointegrate plasmids and spread within pathogens. In this study, we evaluated the possibility of the emergence of a stable *mcr-1-* and *tet*(X4)-coharboring plasmid in E. coli based on *mcr-1*-positive E. coli strain LD91-1 and *tet*(X)-positive E. coli strains RW7-1 and RF10-1. Our findings highlight the potential risk caused by such emerging MDR cointegrate plasmids during bacterial evolution.

## RESULTS

### Construction of plasmids coharboring *mcr-1* and *tet*(X4).

**FIG 1:**
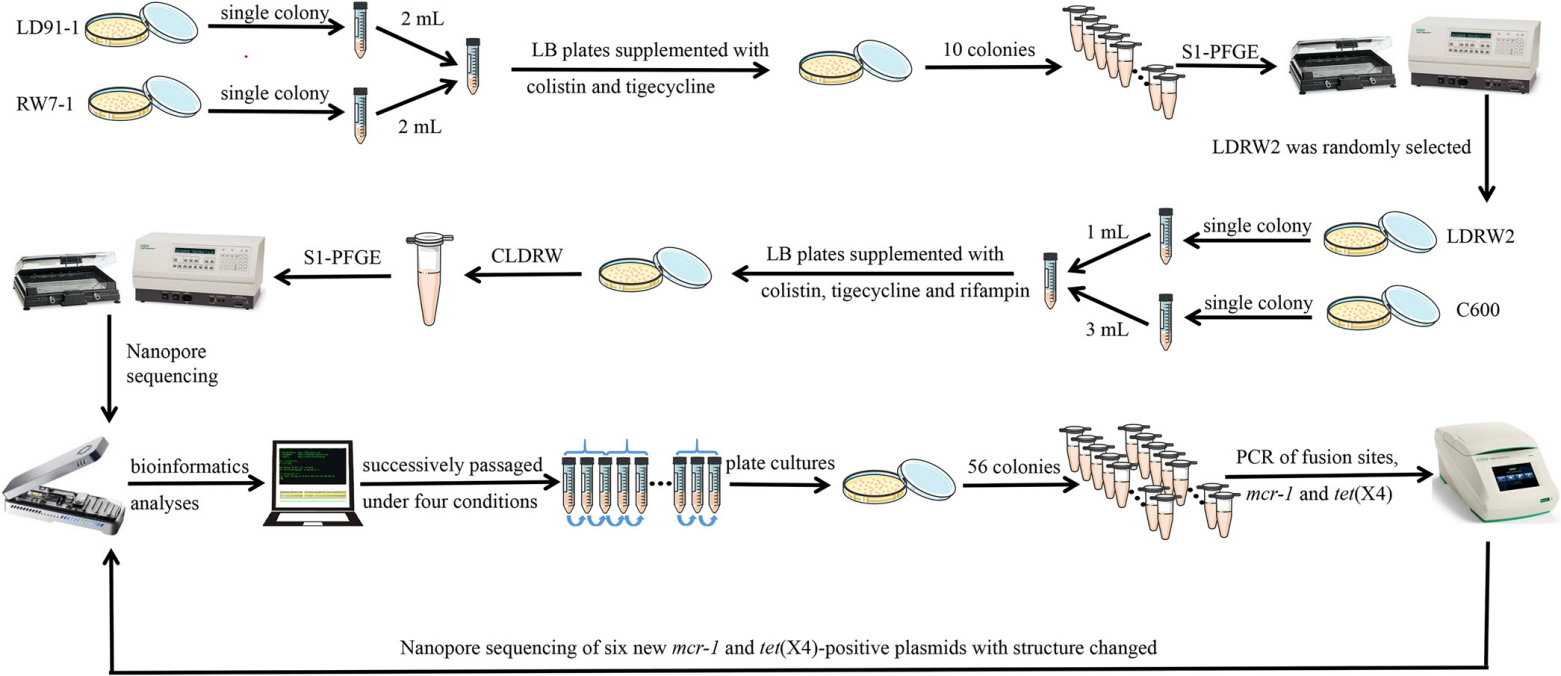
The experiment workflow in the study. Processes include construction of plasmids coharboring *mcr-1* and *tet*(X4), plasmid stability and evolution experiments, cointegrate plasmid sequencing, and bioinformatics analysis.

**FIG 2:**
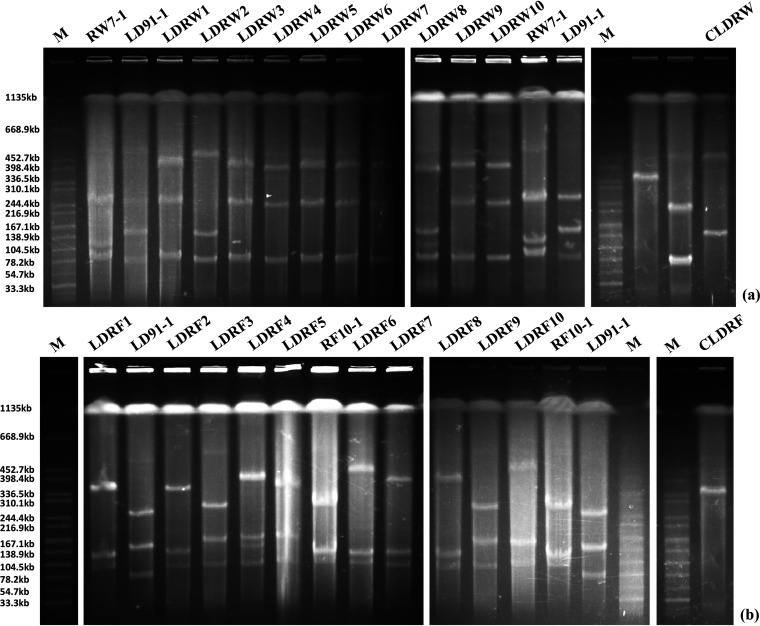
S1-PFGE fingerprints of randomly selected transconjugants carrying *mcr-1* and *tet*(X4). (a) LDRW1 to LDRW10 were the transconjugants of LD91-1 and RW7-1, and CLDRW was the transconjugant of LDRW2 and C600. (b) LDRF1 to LDRF10 were the transconjugants of LD91-1 and RF10-1, and CLDRF was the transconjugant of LDRF4 and C600.

To construct plasmids coharboring *mcr-1* and *tet*(X4), conjugation experiments were conducted, and S1-PFGE and Nanopore sequencing were used to confirm cointegrates. The process is presented in a flowchart in Fig. 1. Twenty transconjugants (LDRW1 to LDRW10 were the transconjugants of LD91-1 and RW7-1, and LDRF1 to LDRF10 were the transconjugants of LD91-1 and RF10-1) carrying *mcr-1* and *tet*(X4) were randomly selected. We designed three specific primer pairs on the chromosomes of E. coli LD91-1, RW7-1, and RF10-1 (Table S1 in the supplemental material). Therefore, we confirmed that transconjugants LDRW1 to LDRW10 were generated by RW7-1 as the donor and LD91-1 as the recipient, and transconjugants LDRF1 to LDRF10 were generated by LD91-1 as the donor and RF10-1 as the recipient. According to S1-PFGE fingerprints, we found that 10 transconjugants from LD91-1 and RW7-1 were harboring a large plasmid of >398 kb that was different from those of their parental strains (Fig. 2a). However, 10 transconjugants from LD91-1 and RF10-1 were relatively complex and diverse (Fig. 2b). We speculated that the structures of some plasmids changed via reorganization in the process of conjugation. To further learn the locations of *mcr-1* and *tet*(X4) and simplify the host factors for *mcr-1-* and *tet*(X4)-cocarrying plasmids, transconjugants LDRW2 and LDRF4 were randomly selected to conduct secondary conjugation experiments with E. coli strain C600. After conjugation of LDRW2 and E. coli C600, an *mcr-1*- and *tet*(X4)-positive transconjugant, CLDRW, was randomly selected to conduct S1-PFGE. The DNA fingerprint showed that CLDRW possessed two plasmids. One was an IncFIB plasmid according to the PCR-based replicon typing, and it had the same size as pLD91-1-146kb (Fig. 2a) (16, 22). We speculated that plasmid pCLDRW_146k came from E. coli LD91-1. The other one was a large plasmid of >398 kb and was possibly a fusion plasmid carrying *mcr-1* and *tet*(X4) (Fig. 2a). After conjugation of LDRF4 and E. coli C600, one *mcr-1-* and *tet*(X4)-positive transconjugant, CLDRF, was randomly selected to conduct S1-PFGE. The fingerprint showed that only one plasmid existed in CLDRF, indicating that the plasmid was possibly a fusion plasmid carrying *mcr-1* and *tet*(X4) (Fig. 2b).

**FIG 3:**
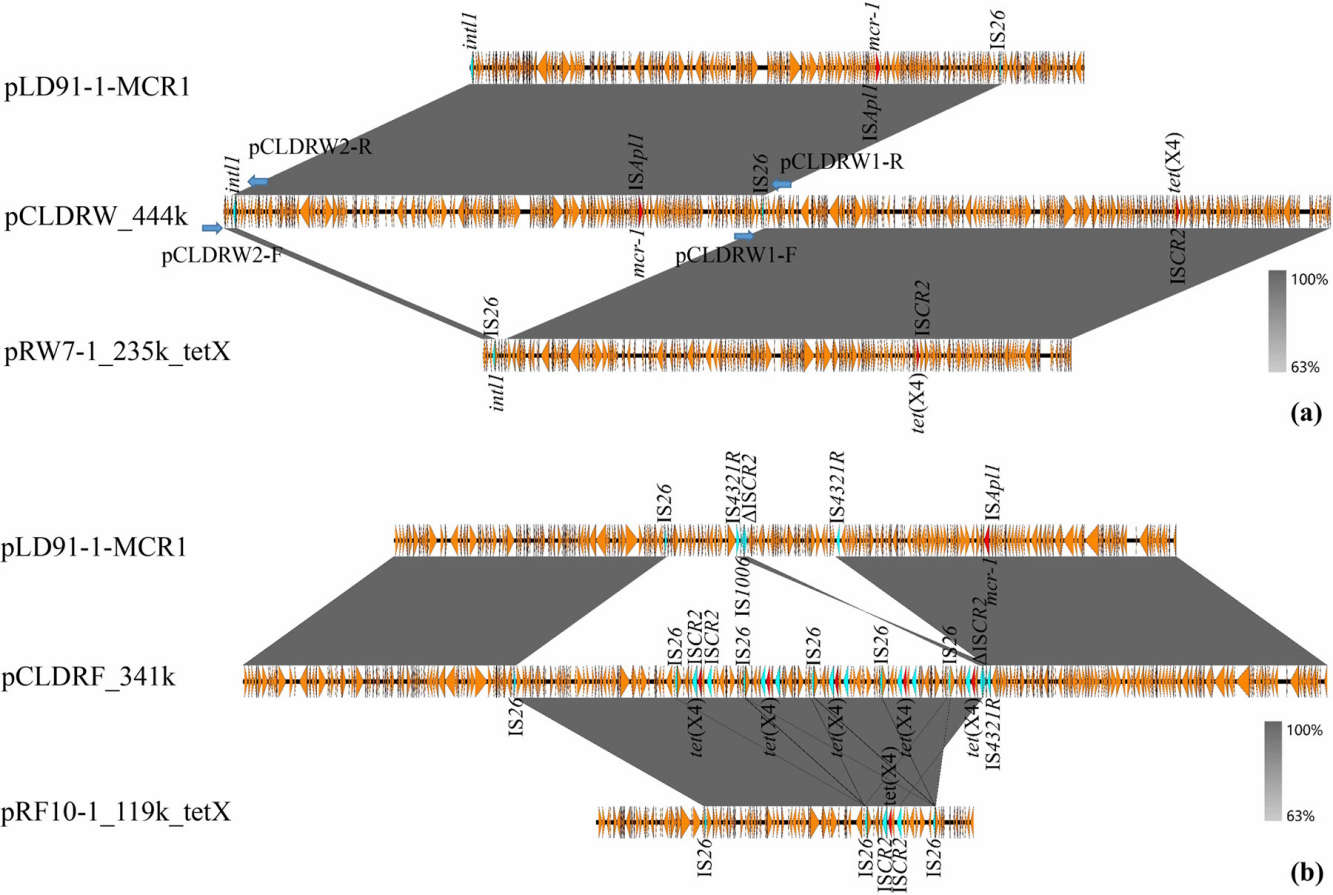
The underlying molecular mechanisms of two constructed *mcr-1-* and *tet*(X4)-coharboring plasmids. (a) Linear sequence alignment among pLD91-1-MCR1, pRW7-1_235_tetX, and pCLDRW_444k. Blue arrows indicate the directions of the specific primers used to screen the fusion sites, and names of primers are marked next to the arrows. (b) Linear sequence alignment among pLD91-1-MCR1, pRF10-1_119k_tetX, and pCLDRF_341k. The regions in gray represent two linked areas with high similarity.

To probe the molecular mechanism of plasmid reorganization, plasmid DNAs of CLDRW and CLDRF were extracted to perform Oxford Nanopore Technologies MinION long-read sequencing. Both plasmids were finished in a complete and circular state. Further analysis verified that strain CLDRW contained plasmid pCLDRW_444k carrying *mcr-1* and *tet*(X4) and plasmid pCLDRW_146k derived from pLD91-1-146kb. pCLDRW_444k, consisting of parts of pLD91-1-MCR1 and pRW7-1_235k_tetX, was a fusion plasmid generated via homologous recombination. The structures of the two fusion sites were integron integrase gene *intI1* and a short sequence, ATTTTATGTACTATGGGATTTAATTCGGGGTAAATCCCATAG (42 bp), that was adjacent to IS*26*-*hyp* on plasmid pLD91-1-MCR1 (Fig. 3a). Strain CLDRF contained an *mcr-1-* and *tet*(X4)-harboring fusion plasmid, pCLDRF_341k. Plasmid analysis confirmed that the fusion event occurred through three ISs: IS*26*, ΔIS*CR2*, and IS*4321*R (Fig. 3b). In addition, five copies of *tet*(X4) were found in pCLDRF_341k, but only one copy of *tet*(X4) was found in the original pRF10-1_119k_tetX (Fig. 3b). Four *tet*(X4)-bearing repeat structures in pCLDRF_341k were IS*26*-flanked segments of 22,362 bp (Fig. 3b). Tandem repeats of *tet*(X4) and other resistance genes mediated by IS*26* have been reported previously (15, 23). One *tet*(X4)-bearing structure was flanked by IS*26* and ΔIS*CR2* that was adjacent to fusion sites, and the ΔIS*CR2* in the structure participated in the fusion of pLD91-1-MCR1 and pRF10-1_119k_tetX (Fig. 3b). The unpredictable tandem repeats may cause difficulties for plasmid evolution research in terms of complex genetic structures, so we chose the strain CLDRW carrying the relatively simple pCLDRW_444k for the following research.

### Plasmid stability and evolution.

**FIG 4:**
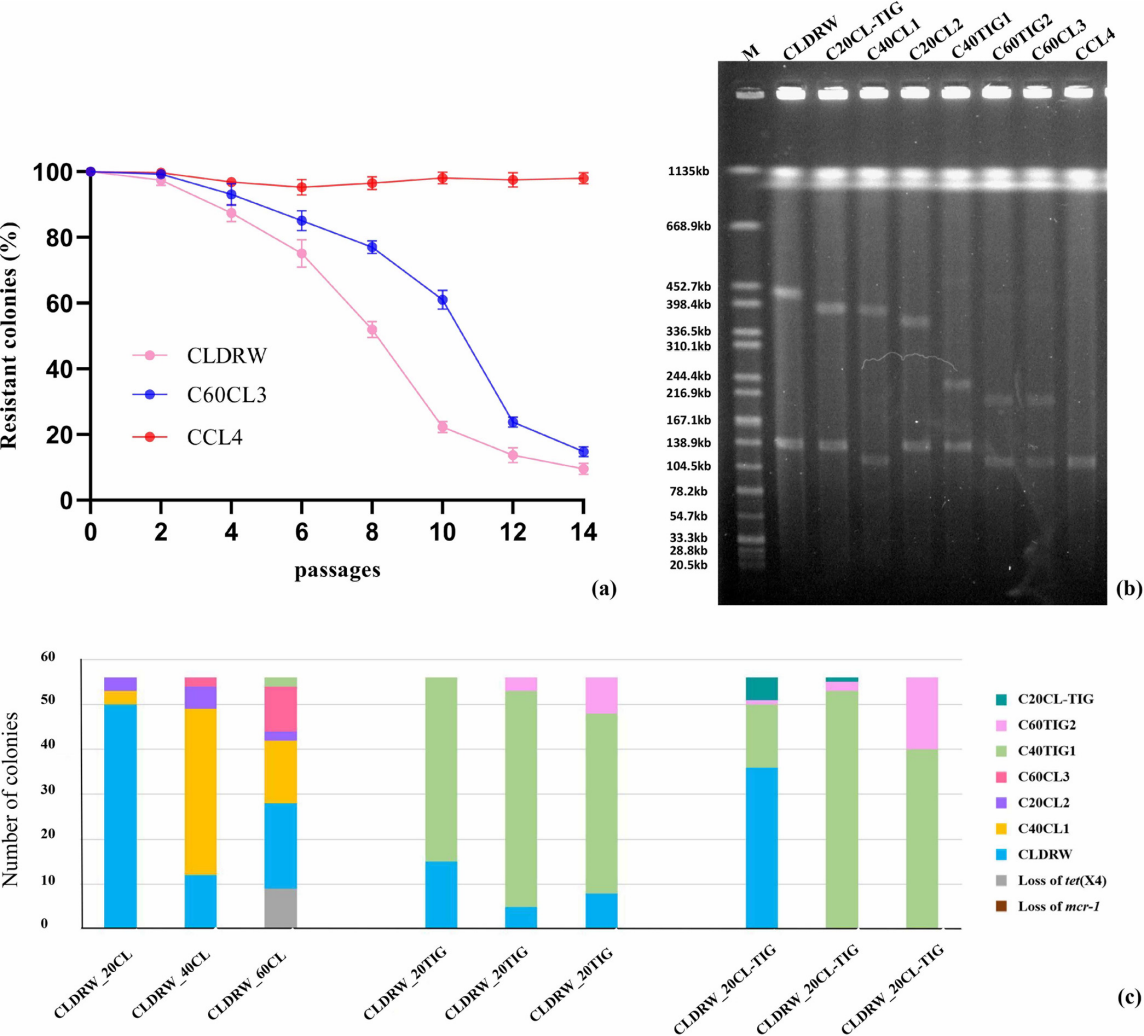
Plasmid stability and evolution. (a) Stability of plasmid pCLDRW_444k in strain CLDRW, pC60CL3_215k in strain C60CL3, and pCCL4_122k in strain CCL4. The frequency of stable plasmids was calculated by the (colonies grown on plate containing colistin and tigecycline / colonies on antibiotic-free LB plate) × 100%. (b) S1-PFGE fingerprints of CLDRW and 7 strains carrying evolved *mcr-1-* and *tet*(X4)-coharboring plasmids. (c) The distribution of different *mcr-1-* and *tet*(X4)-coharboring plasmids in 56 colonies from different antimicrobial environments and passages. ‘CL’ and ‘TIG’ denote colistin pressure and tigecycline pressure, respectively. Numbers ‘20’, ‘40’ and ‘60’ stand for passage 20, passage 40 and passage 60.

The stability of plasmid pCLDRW_444k was determined. Loss of *mcr-1* and *tet*(X4) occurred in CLDRW from day 1 (passage 2) in an antibiotic-free environment (Fig. 4a). We did not further confirm whether the loss of *mcr-1* and *tet*(X4) was based on the loss of plasmid pCLDRW_444k or of the *mcr-1*- and *tet*(X4)-bearing fragments, because the result was enough to show that pCLDRW_444k was extremely unstable in an antibiotic-free environment. However, plasmid transfer and subsequent host adaptation is likely promoted by selection for plasmid-encoded functions, such as antibiotic resistance under the corresponding pressure, so we gained insight into the evolution of the plasmid under antibiotic selection pressure.

In order to analyze the dynamic process of the evolution of plasmid pCLDRW_444k, strain CLDRW carrying pCLDRW_444k was successively passaged under three antibiotic-containing-culture conditions for 30 days. We found six new *mcr-1-* and *tet*(X4)-positive plasmids with changed structures that evolved from pCLDRW_444k (Fig. 4b and Fig. S1). The distribution of seven *mcr-1-* and *tet*(X4)-coharboring plasmids in each of 56 colonies from different antimicrobial environments and passages was monitored by PCR and Sanger sequencing. Specific primers for fusion sites and site structures are described in Table 1.

**TABLE 1:** Specific primers that were used to screen the fusion sites of different *mcr-1*- and *tet*(X4)-coharboring plasmids during the evolutionary processes and the structures of fusion sites^*a*^

Strain	Plasmid	Primer	Sequence (5′–3′)	Size (bp)	Structure of fusion site
CLDRW	pCLDRW_444k	pCLDRW1-F	TAGCTCTGCGCCACATTTCA	580	ATTTTATGTACTATGGGATTTAATTCGGGGTAAATCCCATAG
		pCLDRW1-R	ACAGATGTCGGGTGACCAAC		
		pCLDRW2-F	ATCTACACGACGGGGAGTCA	3,043	*intI1*
		pCLDRW2-R	GATGCCAAAGCGATAGCGTG		
C40CL1	pC40CL1_400k	pCLDRW1-F	TAGCTCTGCGCCACATTTCA	580	ATTTTATGTACTATGGGATTTAATTCGGGGTAAATCCCATAG
		pCLDRW1-R	ACAGATGTCGGGTGACCAAC		
		pC40CL1-R	ACAGGAGTCGGGTTTTGCTC	1,157	Adjacent to IS*26*
		pC40CL1-F	TGCGTATGGCGTCAGGATAG		
C20CL2	pC20CL2_376k	pCLDRW1-F	TAGCTCTGCGCCACATTTCA	580	ATTTTATGTACTATGGGATTTAATTCGGGGTAAATCCCATAG
		pCLDRW1-R	ACAGATGTCGGGTGACCAAC		
		pC20CL2-R	GGCAACTAGCAGTACCAGCA	3,080	ΔISSWi1-IS*26*
		pC20CL2-F	TGCCGAACAGAAACGACAGA		
C60CL3	pC60CL3_215k	pC60CL3-F	AGCCGGTGACTAACAGGTTG	1,701	*hyp*
		pC60CL3-R	CTTCTCAGCAGCCAAAACCG		
		pC40CL1-F	ACAGGAGTCGGGTTTTGCTC	1,157	Adjacent to IS*26*
		pC40CL1-R	TGCGTATGGCGTCAGGATAG		
C40TIG1	pC40TIG1_243k	pCLDRW2-F	ATCTACACGACGGGGAGTCA	3,043	*intI1*
		pCLDRW2-R	GATGCCAAAGCGATAGCGTG		
		pC40TIG1-F	CTTCCTCTTTCGCTTCCGGT	1,069	*hyp*
		pC40TIG1-R	GGCCAACCTCTATACCCTGC		
C60TIG2	pC60TIG2_221k	pC40TIG1-F	CTTCCTCTTTCGCTTCCGGT	1,069	*hyp*
		pC40TIG1-R	GGCCAACCTCTATACCCTGC		
		pC60TIG2-F	AGTAGCGAGGAGGAGTCGTT	1,271	IS*26*
		pC60TIG2-R	CCCCGTGATTGACTGGTTCT		
C20CL-TIG	pC20CL-TIG_406k	pC60TIG2-F	AGTAGCGAGGAGGAGTCGTT	1,271	IS*26*
		pC60TIG2-R	CCCCGTGATTGACTGGTTCT		
		pCLDRW1-F	TAGCTCTGCGCCACATTTCA	580	ATTTTATGTACTATGGGATTTAATTCGGGGTAAATCCCATAG
		pCLDRW1-R	ACAGATGTCGGGTGACCAAC		

a CL, colistin pressure; TIG, tigecycline pressure; 20, 40, and 60, passage 20, passage 40, and passage 60.

Under colistin pressure, the *mcr-1* gene persisted stably during the passages of CLDRW, but *tet*(X4) was lost in nine colonies in passage 60 (Fig. 4c and Table S2). Four new evolved *mcr-1-* and *tet*(X4)-positive plasmids appeared after serial passages (Fig. 4c and Table S2). The proportion of pCLDRW_444k plasmid-carrying cells declined rapidly in passages 40 and 60. Plasmids pC40CL1_400k and pC20CL2_376k emerged in passage 20. Plasmid pC40CL1_400k had a visible increase in passage 40 but was reduced in passage 60. Plasmid pC20CL2_376k kept a low level the whole time. Plasmid pC60CL3_215k emerged in passage 40 and had an obvious increase in passage 60. In addition, two strains carrying pC40TIG1_243k emerged in passage 60; this plasmid was mainly distributed under culture conditions containing tigecycline alone and containing both colistin and tigecycline (Fig. 4c and Table S2).

Under tigecycline pressure, *mcr-1* and *tet*(X4) were stable during passages of CLDRW. Two evolved *mcr-1*- and *tet*(X4)-positive plasmids appeared after serial passages (Fig. 4c and Table S2). The number of pCLDRW-444k containing strains had dropped sharply in passage 20. Plasmid pC40TIG1_243k kept a high level from passage 20 to passage 60. Plasmid pC60TIG2_221k emerged in passage 40 and had a visible increase in passage 60 (Fig. 4c and Table S2).

Under the pressure of colistin and tigecycline, *mcr-1* and *tet*(X4) were stable during passages of CLDRW. Three evolved *mcr-1*- and *tet*(X4)-containing plasmids appeared after serial passages (Fig. 4c and Table S2). In passage 40, plasmid pCLDRW_444k had completely disappeared. Plasmid pC20CL-TIG_406k emerged in passage 20, but it was obviously reduced in passage 40 and had disappeared completely in passage 60. pC40TIG1_243k kept a high level from passage 20 to passage 60. pC60TIG2_221k emerged in passage 20 and had an apparent increase in passage 60 (Fig. 4c and Table S2).

In E. coli strains C40TIG1, C20CL2, and C20CL-TIG, pCLDRW_146k was also coharbored with a *mcr-1*- and *tet*(X4)-positive plasmid (Fig. 4b). In strains C40CL1, C60TIG2, and C60CL3, however, *mcr-1-* and *tet*(X4)-coharboring plasmids were found to coexist with pCLDRW_125k, which was derived from pCLDRW_146k, with 21 kb from pCLDRW_146k being deleted after evolution (Fig. 4b).

These results suggested that pCLDRW-444k was not stable in E. coli C600. Under the pressure of different antimicrobials, the evolutionary process was also different. But the general evolutionary trend was that pCLDRW_444k was gradually getting smaller through removing redundant or metabolically costly genes that increase the burden of plasmid carriage.

### Fitness costs of *mcr-1-* and *tet*(X4)-coharboring plasmids on E. coli C600.

**FIG 5:**
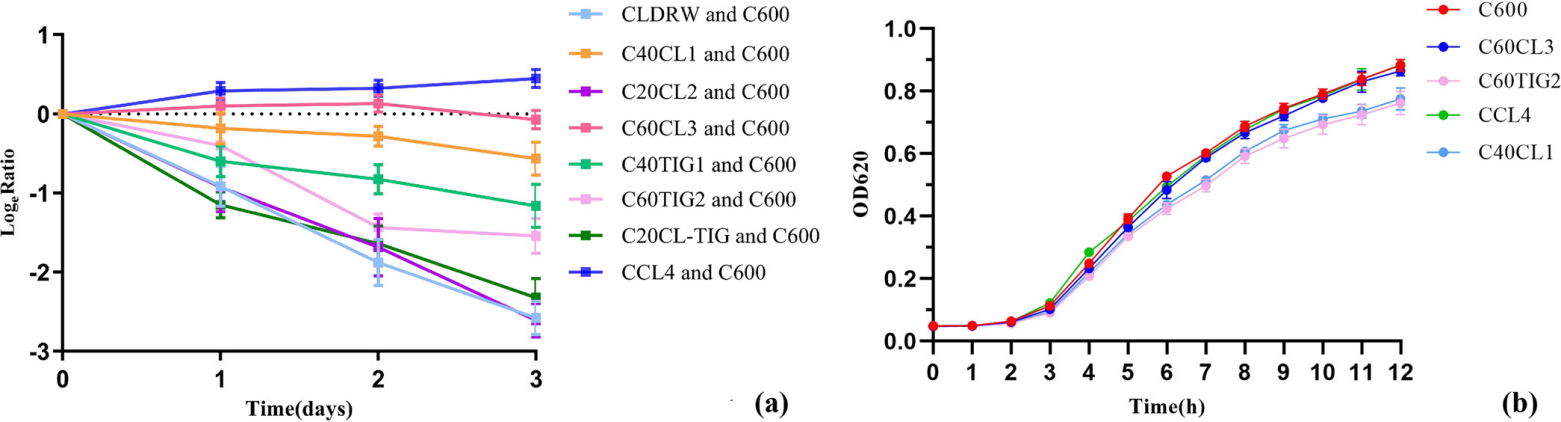
The fitness effects on the E. coli host strain C600 due to the presence of plasmids coharboring *mcr-1* and *tet*(X4). (a) Dynamics of competition experiments between E. coli C600 carrying different *mcr-1-* and *tet*(X4)-coharboring plasmids and E. coli C600. (b) Growth curves of four E. coli C600 strains carrying *mcr-1-* and *tet*(X4)-coharboring plasmids and E. coli C600.

To measure the fitness effects on the host strain E. coli C600 due to the presence of *mcr-1-* and *tet*(X4)-coharboring plasmids, we performed growth curves and competition assays. We first performed competition assays that allow measurement of the relative fitness of two bacteria competing for resources in the same culture. Carriage of pCLDRW_444k, pC20CL-TIG_406k, pC20CL2_376k, pC40TIG1_243k, and pC60TIG2_221k resulted in high fitness costs on E. coli C600 (Fig. 5a). The plasmid pC40CL1_400k imposed a relatively small burden on E. coli C600 compared with the aforementioned plasmids (Fig. 5a). No apparent fitness effects of plasmid pC60CL3_215k were imposed on E. coli C600 (Fig. 5a). Growth curve measurements were conducted for E. coli strains C600, C40CL1 harboring pC40CL1_400k, C60TIG2 harboring pC60TIG2_221k, and C60CL3 harboring pC60CL3_215k. Strains C40CL1, C60TIG2, and C60CL3 also carried pCLDRW_125k. The growth rates of C60TIG2 and C40CL1 were obviously lower than that of E. coli C600 (Fig. 5b), suggesting that carriage of pC40CL1_400k and pC60TIG2_221k imposed a burden on E. coli C600. However, no significant differences in growth rates were observed between strains C60CL3 and C600 (Fig. 5b). These results indicated that plasmid pC60CL3_215k cocarrying *mcr-1* and *tet*(X4) did not have a significant effect on fitness cost.

### Emergence of the stable *mcr-1-* and *tet*(X4)-coharboring plasmid pCCL4_122k.

**FIG 6:**
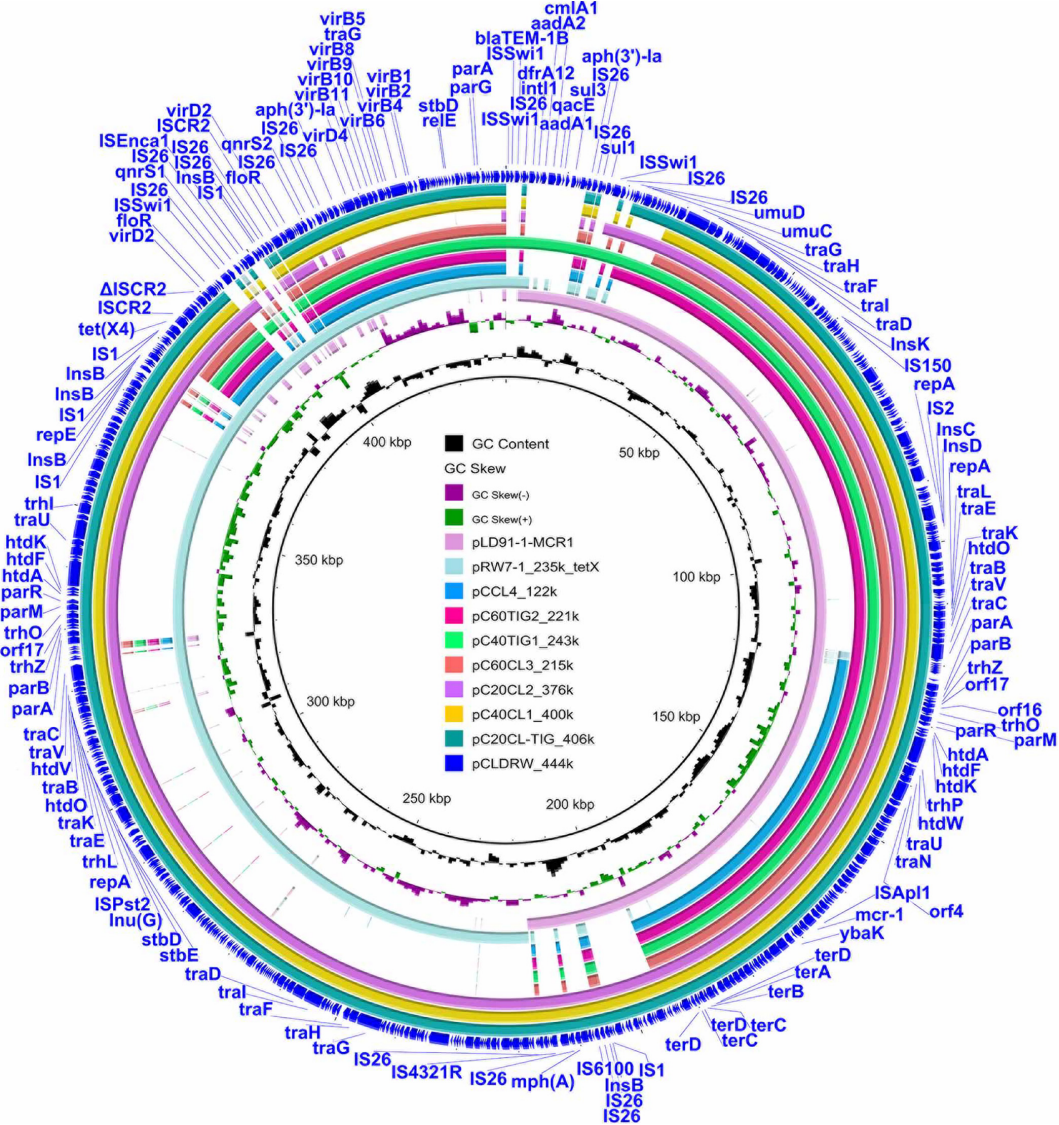
Circular comparisons between the plasmid pCLDRW_444k and its evolved plasmids. The outmost circle indicates the reference plasmid pCLDRW_444k with genes annotated.

Since pC60CL3_215k had no fitness costs on E. coli C600, we continued to study whether this plasmid could exist stably in C600. Strain C60CL3 carrying pC60CL3_215k was successively passaged in an antibiotic-free environment for 7 days (14 passages) and under colistin-containing conditions for 30 days (60 passages). However, *mcr-1* and *tet*(X4) loss occurred in C60CL3 from day 2 (passage 4) in an antibiotic-free environment (Fig. 4a). Under colistin pressure, *mcr-1* and *tet*(X4) were stable during serial passaging of C60CL3. But a novel *mcr-1-* and *tet*(X4)-coharboring plasmid, pCCL4_122k, appeared in passage 60 (in 31 of 56 colonies) (Fig. 6). Stability and fitness assessments of pCCL4_122k in CCL4 were conducted. More than 95% of colonies still contained pCCL4_122k in passage 14 (Fig. 4a), suggesting that a relatively stable plasmid, pCCL4_122k, had successfully evolved from pC60CL3_215k. No obvious difference in growth was observed between E. coli strains CCL4 and C600 after a 12-h growth assessment (Fig. 5b). Competition assays of E. coli CCL4 against E. coli C600 showed that a slight fitness increase was observed in CCL4 carrying pCCL4_122k (Fig. 5a). Meanwhile, pCCL4_122k could transfer from E. coli CCL4 to E. coli strain J53 at a frequency of (8.98 ± 0.4) × 10^−7^, which may be correlated with the increased fitness effects of pCCL4_122k in E. coli C600 (24). These results suggested that compensatory adaptation had occurred during evolution to overcome the cost associated with pCLDRW_444k carriage. And one of the ways that plasmid pCLDRW_444k evolved into a stable plasmid was through removing redundant genes responsible for the same functions, such as gene clusters encoding plasmid conjugative transfer proteins (Fig. 6). In addition, the evolution of pCLDRW_444k was closely related to IS*26* (Table 1 and Fig. S1), which may facilitate the formation of various plasmid types and accelerate the evolution of MDR plasmids (25).

## DISCUSSION

Conjugative plasmids have contributed to the rapid spreading of antimicrobial resistance genes among clinically important pathogens (26, 27). Fusion between plasmids with different Inc types occurs frequently, which could further extend the resistance profiles of pathogens and broaden the host spectrum of plasmids. Recent years have seen various ISs reported to participate in the formation of cointegrate plasmids (16, 28, 29). *mcr-1* and *tet*(X4) were predominantly found on MDR plasmids carrying various ISs (6, 7, 15). The formation of the fusion plasmids coharboring *mcr-1* and *tet*(X4) is possible. Based on *mcr-1*-positive E. coli strain LD91-1 and *tet*(X4)-harboring E. coli strains RW7-1 and RF10-1, we proved this hypothesis. In this study, we investigated the formation and dynamic evolution of *mcr-1-* and *tet*(X4)-coharboring cointegrate plasmids. The generation of fusion plasmids pCLDRW_444k and pCLDRF_341k suggested that the presence of sequences *intI1*, IS*26*, ΔIS*CR2* and IS*4321*R in two plasmids could facilitate plasmid fusion and coevolution of both critical resistance genes.

Exposure to antimicrobials could lead to the rapid transmission of resistance genes in conjugative plasmids, as well as promoting the evolution of MDR plasmids. In our study, a stable *mcr-1-* and *tet*(X4)-coharboring plasmid, pCCL4_122k, appeared in E. coli over a long evolutionary period under the selection pressure of colistin. pCCL4_122k had greater stability and a significant increase in fitness compared with other *mcr-1-* and *tet*(X4)-coharboring plasmids, indicating that it was more capable of being transferred by conjugation and persisting within the host bacteria and consequently causing the rapid dissemination of *mcr-1* and *tet*(X4) among bacteria. The plasmid evolutionary process described in our study provides an example of how novel *mcr-1-* and *tet*(X4)-coharboring plasmids with characteristics that help them spread widely can emerge during successive passages. In addition, we noticed that the evolution process of plasmid pCLDRW_444k under different selection pressures was distinct. These findings emphasize the importance of the rational use of antimicrobials to inhibit the formation and evolution of cointegrate plasmids.

All the replicons of pLD91-1-MCR1 (IncHI2 and IncN) and pRW7-1_235k_tetX [IncFIA(HI1), IncHI1A, IncHI1B(R27), and IncX1] appeared in cointegrate plasmid pCLDRW_444k, but plasmid pC60CL3_215k, originating from pCLDRW_444k, harbored replicons IncHI2, IncFIA(HI1), and IncX1. Plasmid pCCL4_122k only contained replicon IncX1. pCLDRW_444k and pC60CL3_215k were unstable. It has been reported that *tet*(X4) often locates on multireplicon plasmids, including pRW7-1_235k_tetX and pRF10-1_119k_tetX (15). It is worth thinking about whether this multireplicon phenomenon is related to the cointegration of plasmids during bacterial evolution. In this study, plasmids pC20CL-TIG_406k, pC40CL1_400k, and pC20CL2_376k included large regions of pLD91-1-MCR1 and pRW7-1_235k_tetX (Fig. 6). However, plasmids pC40TIG1_243k, pC60TIG2_221k, and pC60CL3_215k only consisted of a large region of pLD91-1-MCR1 and a relatively small fragment of pRW7-1_235k_tetX after further evolution (Fig. 6). We also conducted an auxiliary experiment, stability tests of pLD91-1-MCR1 and pRW7-1_235k_tetX, and the results showed that pLD91-1-MCR1 was more stable than pRW7-1_235k_tetX (data not shown). One of the reasons for this phenomenon may be the complexity of the replicons in plasmid pRW7-1_235k_tetX. These issues warrant further investigation.

A limitation of this study is that no more transconjugant experiments with *mcr-1*-positive strains and *tet*(X4)-positive strains were conducted to evaluate the evolution and destiny of *mcr-1*- and *tet*(X4)-coharboring plasmids. Investigation of the evolution of *mcr-1-* and *tet*(X4)-coharboring plasmids was mainly based on PCR of fusion sites in numerous strains. This may overlook some plasmids that have the same fusion sites but still have different structures. In addition, we did not successfully evaluate the frequency of the formation of *mcr-1-* and *tet*(X4)-coharboring cointegrate plasmids, because the final confirmation of these plasmids requires costly Nanopore long-read sequencing and then is not suitable for mass sequencing. However, we noticed that the formation of recombinant plasmids is almost 100% according to S1-PFGE fingerprints of LDRW1 to LDRW10 and LDRF1 to LDRF10 (Fig. 2), which still poses a great threat for the dissemination of resistance genes and needs attention.

In conclusion, we describe the generation of different *mcr-1-* and *tet*(X4)-coharboring plasmids based on field strains through *in vitro* assays, thus providing important insight into the contributions of *intI1*, IS*26*, ΔIS*CR2*, and IS*4321*R to the generation of cointegrate plasmids carrying *mcr-1* and *tet*(X4) by homologous recombination. Furthermore, a stable *mcr-1-* and *tet*(X4)-coharboring plasmid, pCCL4_122k, evolved from pCLDRW_444k in E. coli over a long evolutionary period under exposure to antimicrobials, which provides direct evidence of plasmid evolution and highlights the importance of antimicrobials in the evolution and diversity of *mcr-1-* and *tet*(X4)-coharboring plasmids. This will accelerate the transmission of *mcr-1* and *tet*(X4) among bacteria, and the rational use of antimicrobials should be promoted to inhibit the formation and evolution of cointegrate plasmids harboring emerging novel resistance genes. In order to avoid the formation of *mcr-1-* and *tet*(X4)-coharboring plasmids, continuous surveillance of the emergence of *mcr-1*-carrying MDR plasmids and *tet*(X4)-bearing MDR plasmids in single bacterial isolates should be implemented.

## MATERIALS AND METHODS

### Bacterial strains and growth conditions.

E. coli LD91-1 is an *mcr-1*-positive strain harboring three plasmids, pLD91-1-MCR1 (246,716 bp), pLD91-1-146kb (146,133 bp), and pLD91-1-76kb (76,292 bp). The *mcr-1* gene is in plasmid pLD91-1-MCR1, which is a typical IncHI2-IncN *mcr-1*-bearing plasmid carrying various resistance genes dispersed among ISs (16). E. coli RW7-1 is a *tet*(X4)-positive strain harboring three plasmids, pRW7-1_235k_tetX (235,947 bp), pRW7-1_111k (111,494 bp), and pRW7-1_81k (81,582 bp). The *tet*(X4) gene is located on pRW7-1_235k_tetX, which is an MDR plasmid with various ISs and diverse replicons, including IncFIA(HI1), IncHI1A, IncHI1B(R27), and IncX1 (15). E. coli RF10-1 is a *tet*(X4)-harboring strain carrying three plasmids, pRF10-1_269k (269,721 bp), pRF10-1_119k_tetX (119,011 bp), and pRF10-1_98k (97,999 bp) (15). pRF10-1_119k_tetX is a *tet*(X4)-bearing plasmid carrying various ISs and multiple replicons, including IncFIB(K), IncFIA(HI1), and IncX1. E. coli strains LD91-1, RW7-1, and RF10-1, exhibiting plasmid reorganization during conjugation (15, 16), were chosen purposefully to construct plasmids coharboring *mcr-1* and *tet*(X4). The typical bacterial growth media used in this study were LB broth and agar. Given the significance and risk of resistance genes *mcr-1* and *tet*(X4), we adhered to strict biosafety procedures during the experiments. All consumables in contact with bacteria were strictly autoclaved, including bacterial cultures, pipette tips, centrifuge tubes, and so on.

### Construction of plasmids coharboring *mcr-1* and *tet*(X4).

The *mcr-1*-bearing E. coli LD91-1 and two *tet*(X4)-positive strains, RW7-1 and RF10-1, were streaked onto LB agar plates, followed by inoculation into LB broth at 37°C until the cultures reached 0.5 McFarland standard. Then, cultures of RW7-1 and RF10-1 were each mixed with LD91-1 at a ratio of 1:1 and 0.1-ml amounts of the mixed cultures were applied onto LB agar plates, followed by incubation at 37°C for 16 to 20 h. After incubation, we collected the bacterial cultures on plates and diluted them in sterile saline. LB agar plates supplemented with colistin (2 mg/liter) and tigecycline (2 mg/liter) were used to recover transconjugants coharboring *mcr-1* and *tet*(X4). The presence of *mcr-1* and *tet*(X4) was confirmed by PCR using primers as previously described (12, 30). To learn the plasmid profiles of *tet*(X4)- and *mcr-1*-positive transconjugants, S1 nuclease (TaKaRa, Osaka, Japan) pulsed-field gel electrophoresis (PFGE) was performed with the CHEF Mapper XA system (Bio-Rad, CA, USA), and Salmonella Braenderup H9812 restricted with XbaI (TaKaRa, Osaka, Japan) was used as the marker to recognize the size of plasmids (31). According to the plasmid profiles, two transconjugants with *mcr-1-* and *tet*(X4)-cocarrying plasmids were randomly selected to conduct secondary conjugation experiments with E. coli C600 (bearing resistance to rifampin), in order to simplify and unify the bacterial host of *mcr-1-* and *tet*(X4)-cocarrying plasmids. Briefly, cultures of selected *mcr-1-* and *tet*(X4)-cocarrying strains and E. coli C600 were mixed at a ratio of 1:3, followed by incubation on LB agar plates at 37°C for 16 to 20 h. Subsequently, we collected the bacteria on plates and screened for transconjugants on LB agar plates containing colistin (2 mg/liter) and tigecycline (2 mg/liter) in combination with rifampin (300 mg/liter). Then, we identified transconjugants carrying *mcr-1* and *tet*(X4) by PCR. S1-PFGE was again used to verify the size of plasmids in transconjugants.

### Plasmid stability and evolution experiments.

Plasmid stability and evolution experiments were conducted as previously described with minor changes (32). In brief, strains were grown overnight at 37°C in 5 ml LB broth. Then, amounts of 5 μl of the cultures were transferred into 5 ml LB broth under four culture conditions: colistin (2 mg/liter) alone, tigecycline (2 mg/liter) alone, both colistin (2 mg/liter) and tigecycline (2 mg/liter), and antibiotic free. Serial passages of 5-μl amounts of cultures to 5 ml LB broth were performed every 12 h. Periodically, the cultures were passaged for 30 days (60 passages), which corresponds to approximately 600 generations of bacterial growth by the end of the experiment. To assess the plasmid stability, cultures of passages 2, 4, 6, 8, 10, 12, and 14 in antibiotic-free environments were serially diluted in 0.9% saline and plated onto antibiotic-free LB plates and LB plates supplemented with colistin (2 mg/liter) and tigecycline (2 mg/liter). The frequency of stable plasmids was calculated by the (colonies grown on LB plate containing colistin and tigecycline / colonies on antibiotic-free LB plate) × 100%. To learn the process of plasmid evolution in antibiotic-containing conditions, cultures of passages 20, 40, and 60 were inoculated onto antibiotic-free LB plates and cultured overnight at 37°C. Then, 56 colonies from each plate were randomly selected to screen for the presence of *mcr-1*, *tet*(X4), and fusion sites by PCR.

### Competition experiments.

Competition experiments were conducted as previously described with minor changes (33). Overnight cultures from single colonies of E. coli C600 carrying different plasmids and their parental strain E. coli C600 were prepared. Then, samples of E. coli C600 carrying plasmids were mixed in equal proportions with plasmid-free E. coli C600. A volume of 0.05 ml of mixed competitors was transferred into 5 ml fresh LB broth (time point zero), and then incubated at 37°C. After 24 h of growth, 0.05-ml amounts of cultures were transferred into in 5 ml fresh LB broth and further incubated. A total of three transfers (days 1, 2, and 3) were performed for each test series. The concentrations were determined by plating serial dilutions on antibiotic-free LB agar plates and selective agar plates containing 2 mg/liter colistin and 2 mg/liter tigecycline. log_e_ Ratio was calculated using the log_e_ Ratio = log_e_ R(t) − log_e_ R(0), where *R* is the ratio of the concentration of plasmid-carrying and plasmid-free cells in the two competing cultures, *t* is time in days. If there is no difference in fitness between the competing strains, an expected value of log_e_ Ratio = 0 will be acquired. If plasmid carriage reduces host fitness relative to that of plasmid-free E. coli C600, log_e_ Ratio is negative, and it is positive if plasmid carriage improves host fitness. All experiments were performed in triplicate.

### Measurement of growth curves.

Overnight cultures from single colonies of E. coli C600 carrying different plasmids and E. coli C600 were adjusted to 0.5 McFarland standard. Then, 5-μl amounts of adjusted cultures were diluted into 5 ml LB broth and incubated at 37°C, 200 rpm for 12 h. Bacterial growth was monitored by measuring the optical density at 620 nm (OD_620_) using a Multiskan FC microplate photometer (Thermo Fisher Scientific) every 1 h for 12 h at 37°C (34). Experiments were repeated in three separate assays.

### Cointegrate plasmid sequencing and bioinformatics analysis.

Strains were cultured overnight in 100 ml LB broth, and plasmids coharboring *mcr-1* and *tet*(X4) were extracted using the Qiagen plasmid midi kit (Qiagen, Germany). To obtain the complete sequences, plasmid DNA samples were sequenced by using the Oxford Nanopore Technologies MinION long-read sequencing platform as the reported method (35). *De novo* assembly of plasmids by MinION sequencing data was performed with Flye (36). All cointegrate plasmids finished in complete and circular forms were further corrected according to the sequences of the parental plasmids (pLD91-1-MCR1, accession number CP042587; pRW7-1_235k_tetX, accession number MT219825; and pRF10-1_119k_tetX, accession number MT219823) to avoid indel errors generated by long-read data. The Subsystem Technology annotation website server (https://rast.nmpdr.org/rast.cgi) was then used to annotate the plasmids (37). ResFinder 4.1 (38) and PlasmidFinder 2.1 (39) were utilized to assemble and characterize the plasmids (https://cge.cbs.dtu.dk/services/). BRIG and Easyfig were used to generate and visualize the comparisons of plasmids and genetic arrangements (40, 41).

### Data availability.

The sequences of two fusion plasmids, pCLDRW_444k and pCLDRF_341k, carrying *mcr-1* and *tet*(X4), in combination with seven evolved plasmids carrying *mcr-1* and *tet*(X4) from pCLDRW_444k, have been deposited in figshare (https://doi.org/10.6084/m9.figshare.15176184.v1).
