# Supplementary material for: Emerging Opportunity and Destiny of mcr-1- and tet(X4)-Coharboring Plasmids in Escherichia coli
Source: Microbiol Spectr. 2021 Dec 8;9(3):e01520-21. doi: 10.1128/Spectrum.01520-21 (PMC8653826; doi:10.1128/Spectrum.01520-21)
Supplement: SUPPLEMENTAL FILE 1 — Supplemental material. Download SPECTRUM01520-21_Supp_1_seq11.pdf, PDF file, 1.0 MB [file spectrum01520-21_supp_1_seq11.pdf]

Supplementary Materials

Table S1 Specific primers that were used to confirm the donors and recipients in the conjugation process of LD91-1 with RW7-1 and RF10-1.

Strains	Primers	Sequence(5’-3’)	Size (bp)
LD91-1	LD91-1-F	GCCGGGAGCGAAAATCATTC	916
	LD91-1-R	ATCGCGAAATTTAGCACCGC	
RW7-1	RW7-1-F	GAACTCGCACGGACTCATCA	472
	RW7-1-R	CGGAGGTGCAGCATGAGTAT	
RF10-1	RF10-1-F	CATTCTGGGTGCGCGTATTG	536
	RF10-1-R	TATCGCCGAGAGTCGAAAGC	

Table S2 The colony numbers of different *mcr-1* and *tet*(X4) co-harboring plasmids in 56 colonies in different antimicrobial environments and passages.

Passage and pressure	Loss of *mcr-1*	Loss of *tet*(X4)	pCLDRW_444k	pC40CL1_400k	pC20CL2_376k	pC60CL3_215k	pC40TIG1_243k	pC60TIG2_221k	pC20CL-TIG_406k
CLDRW_20CL	0	0	50	3	3	0	0	0	0
CLDRW_40CL	0	0	12	37	5	2	0	0	0
CLDRW_60CL	0	9	19	14	2	10	2	0	0
CLDRW_20T	0	0	15	0	0	0	41	0	0
CLDRW_40T	0	0	5	0	0	0	48	3	0
CLDRW_60T	0	0	8	0	0	0	40	8	0
CLDRW_20CL-TIG	0	0	36	0	0	0	14	1	5
CLDRW_40CL-TIG	0	0	0	0	0	0	53	2	1
CLDRW_60CL-TIG	0	0	0	0	0	0	40	16	0

Note. ‘CL’ and ‘TIG’ denote colistin pressure and tigecycline pressure, respectively. Numbers ‘20’, ‘40’ and ‘60’ stand for passage 20, passage 40 and passage 60.

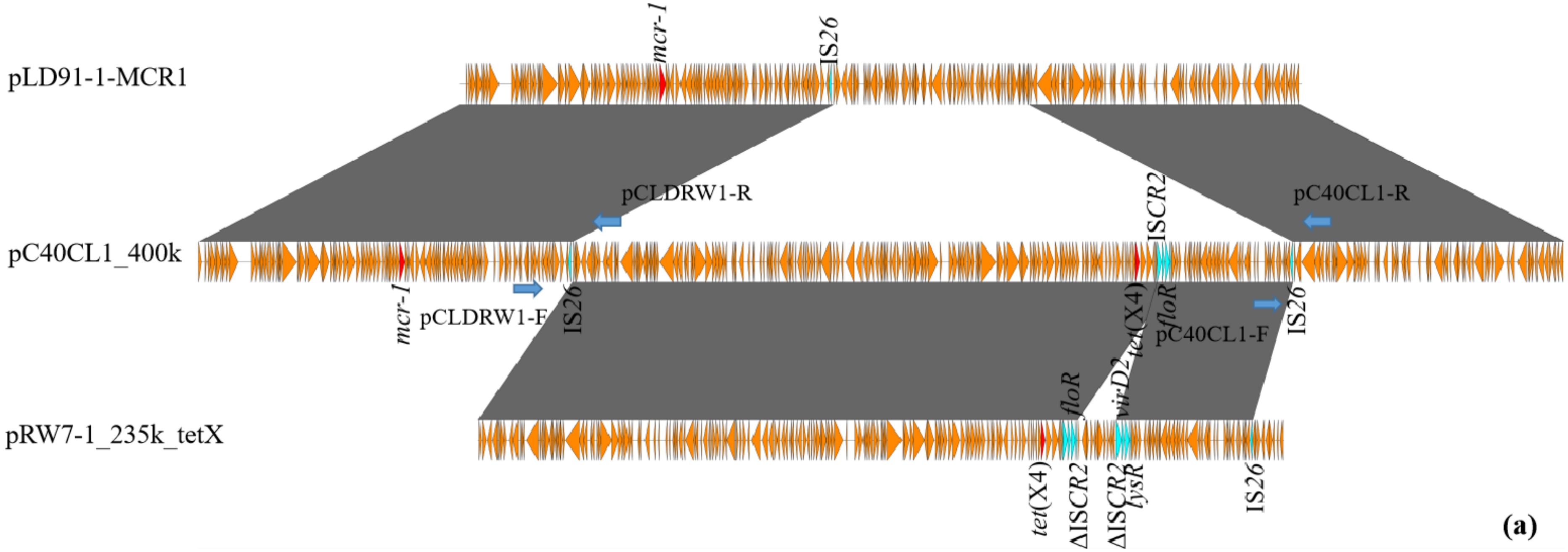

pLD91-1-MCR1
mcr-1
IS26
pC40CL1_400k
pCLDRW1-R
ISCR2
pC40CL1-R
mcr-1
pCLDRW1-F
IS26
tet(X4)
floR
pC40CL1-F
IS26
pRW7-1_235k_tetX
floR
virD2
tet(X4)
ΔISCR2
ΔISCR2
lysR
IS26

(a)

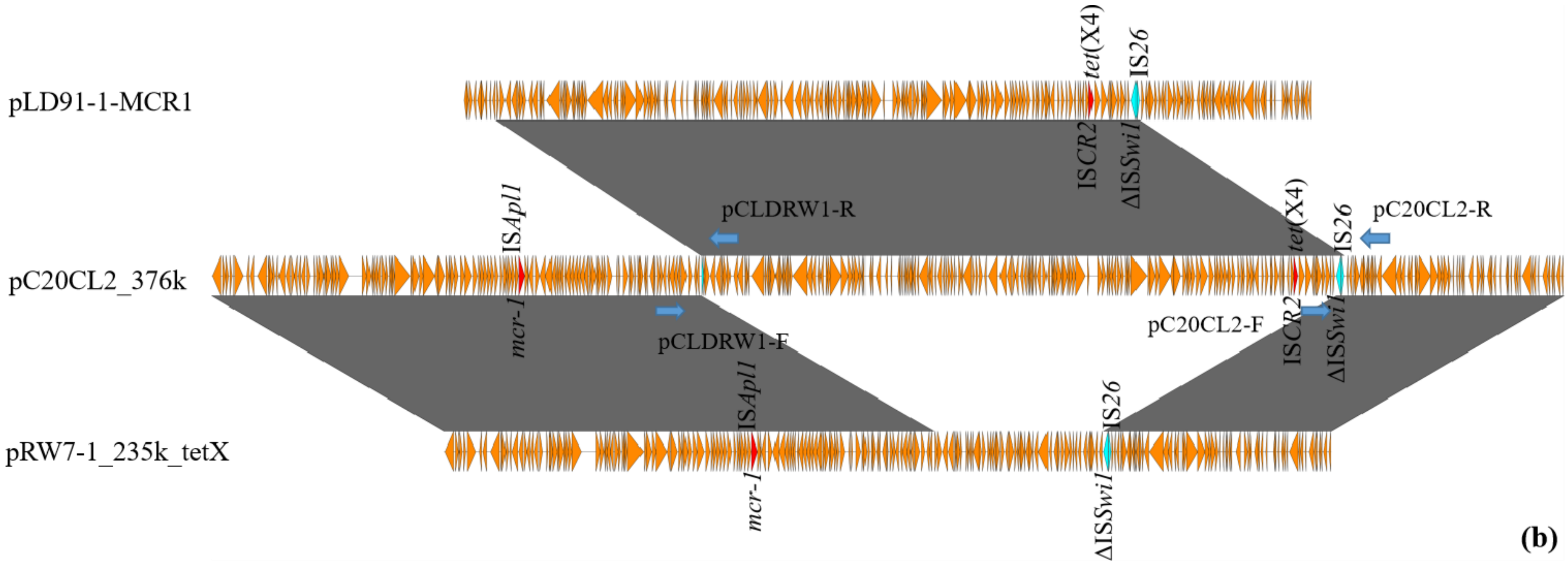
pLD91-1-MCR1
tet(X4)
IS26
ISCR2
ΔISSwi1
pC20CL2_376k
ISApl1
mcr-1
pCLDRW1-R
pCLDRW1-F
tet(X4)
IS26
pC20CL2-R
pC20CL2-F
ISCR2
ΔISSwi1
pRW7-1_235k_tetX
ISApl1
mcr-1
IS26
ΔISSwi1
(b)

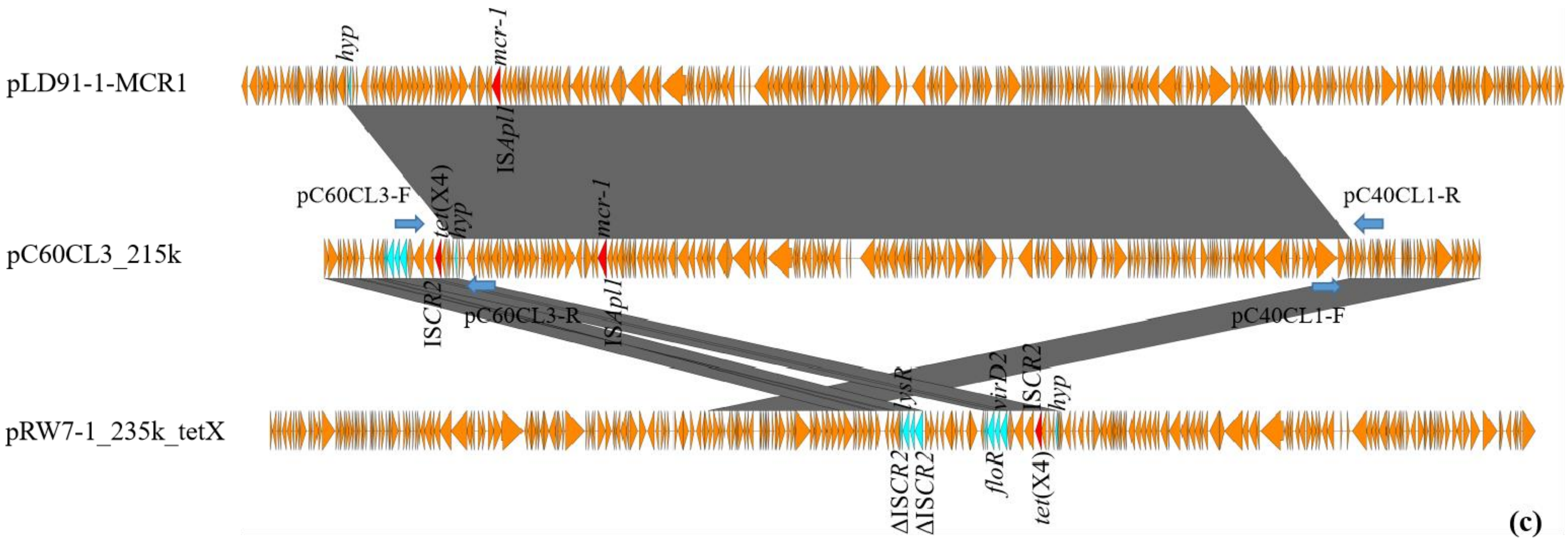
pLD91-1-MCR1
hyp
mcr-1
ISApl1
pC60CL3-F
tet(X4)
hyp
mcr-1
pC40CL1-R
pC60CL3_215k
ISCR2
pC60CL3-R
ISApl1
pC40CL1-F
lysR
virD2
ISCR2
hyp
pRW7-1_235k_tetX
ΔISCR2
ΔISCR2
floR
tet(X4)
(c)

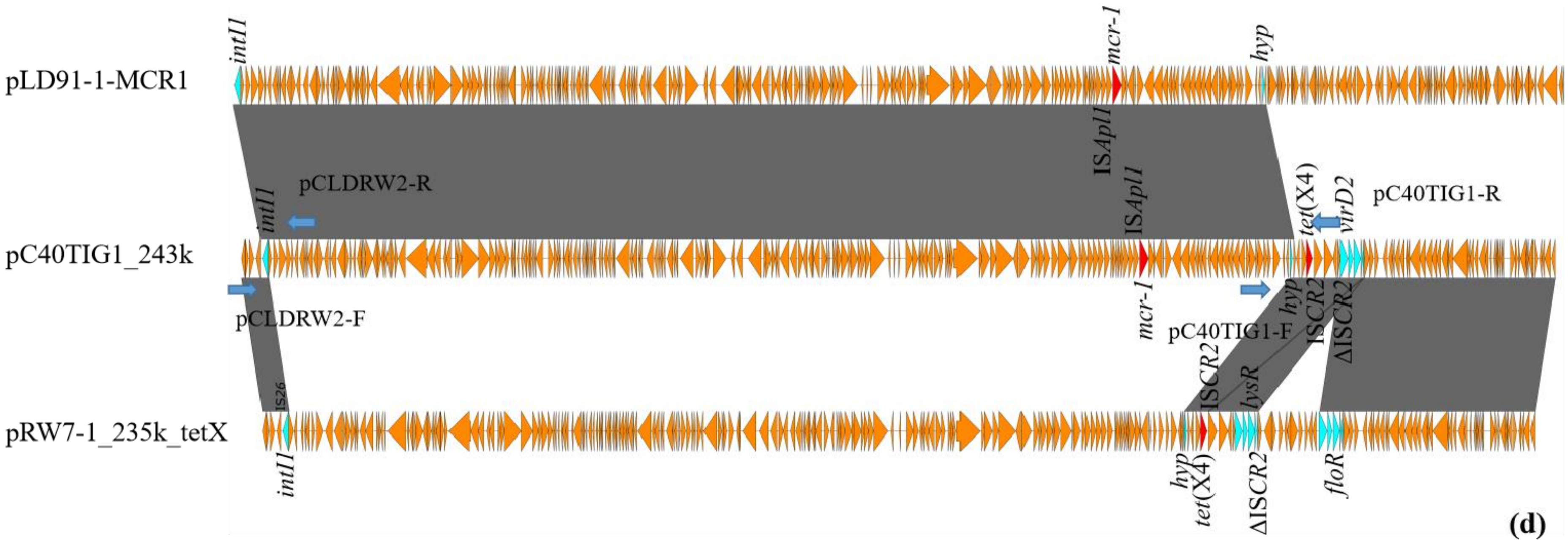

pLD91-1-MCR1
intI1
mcr-1
hyp
ISApl1
pCLDRW2-R
intI1
ISApl1
tet(X4)
virD2
pC40TIG1-R
pC40TIG1_243k
pCLDRW2-F
mcr-1
pC40TIG1-F
hyp
ISCR2
ΔISCR2
ISCR2
lysR
IS26
pRW7-1_235k_tetX
intI1
hyp
tet(X4)
ΔISCR2
floR
(d)

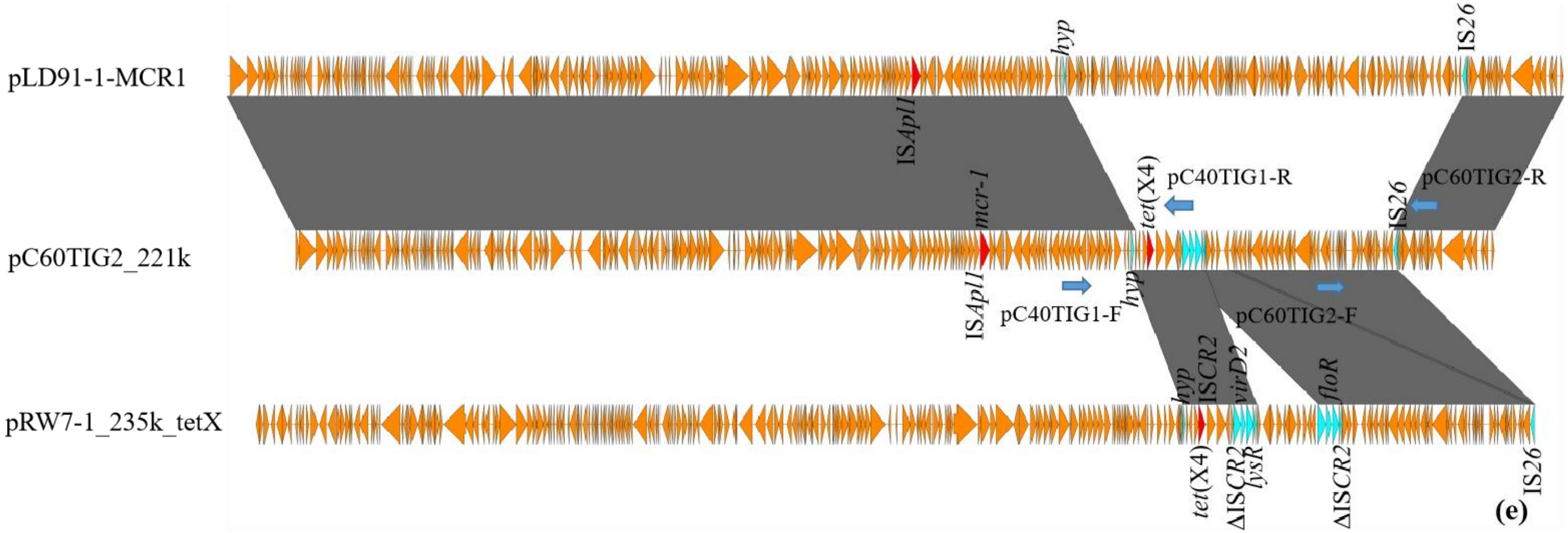
pLD91-1-MCR1
hyp
IS26
ISApl1
mcr-1
tet(X4)
pC40TIG1-R
IS26
pC60TIG2-R
pC60TIG2_221k
ISApl1
pC40TIG1-F
hyp
pC60TIG2-F
hyp
ISCR2
virD2
floR
pRW7-1_235k_tetX
tet(X4)
ΔISCR2
lysR
ΔISCR2
IS26
(e)

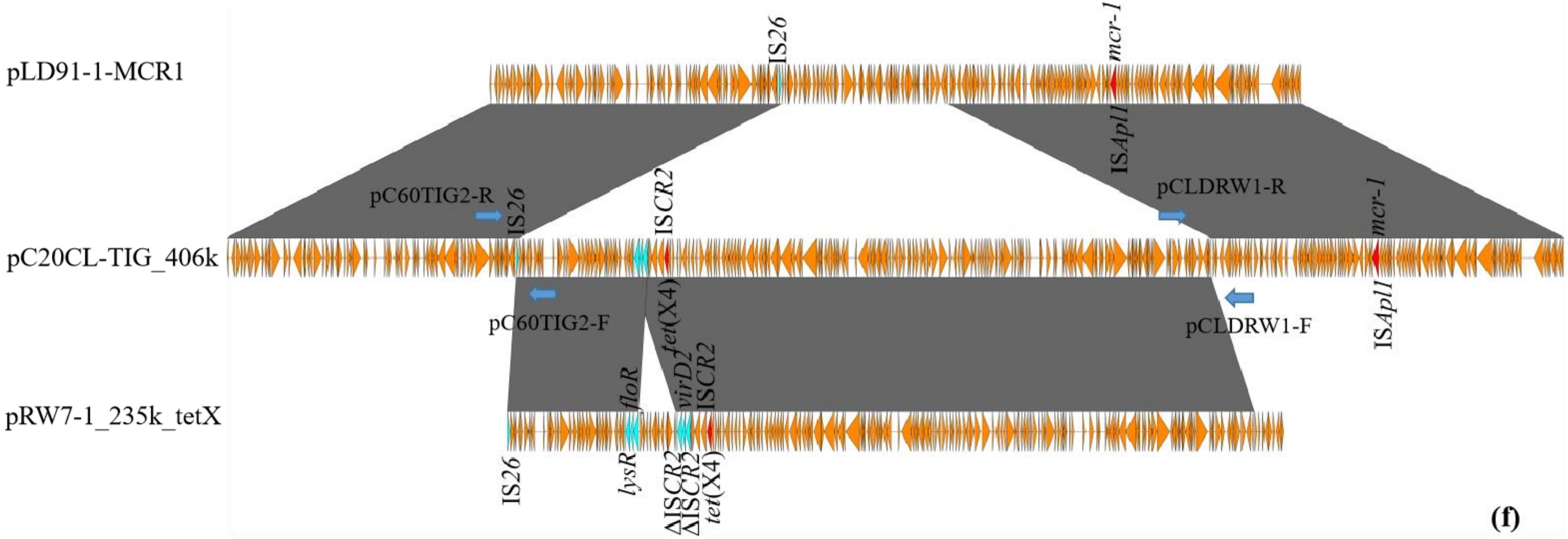

Fig. S1 Linear sequence alignment among pLD91-1-MCR1, pRW7-1_235_tetX and *mcr-1* and *tet*(X4) co-harboring plasmids. (a) Sequence alignment among pLD91-1-MCR1, pRW7-1_235_tetX and pC40CL1_400k. (b) Sequence alignment among pLD91-1-MCR1, pRW7-1_235_tetX and pC20CL2_376k. (c) Sequence alignment among pLD91-1-MCR1, pRW7-1_235_tetX and pC60CL3_215k. (d) Sequence alignment among pLD91-1-MCR1, pRW7-1_235_tetX and pC40TIG1_243k. (e) Sequence alignment among pLD91-1-MCR1, pRW7-1_235_tetX and pC60TIG2_221k. (f) Sequence alignment among pLD91-1-MCR1, pRW7-1_235_tetX andpC20CL-TIG_406k. The regions in gray represent the two linked areas with high similarity. Blue arrows indicate the directions of the specific primers used to screen the fusion sites, and names of primers are marked next to the arrows.
